# Analyzing disparities in COVID-19 testing trends according to risk for COVID-19 severity across New York City

**DOI:** 10.1186/s12889-021-11762-0

**Published:** 2021-09-21

**Authors:** Wil Lieberman-Cribbin, Naomi Alpert, Raja Flores, Emanuela Taioli

**Affiliations:** 1 Institute for Translational Epidemiology and Department of Population Health Science and Policy, Icahn School of Medicine at Mount Sinai, One Gustave L. Levy Place, Box 1133, New York, NY 10029 USA; 2 Department of Thoracic Surgery, Icahn School of Medicine at Mount Sinai, One Gustave L. Levy Place, New York, NY 10029 USA

**Keywords:** Coronavirus; racial disparities, Disaster preparedness, Public health response

## Abstract

**Background:**

Given the interplay between race and comorbidities on COVID-19 morbidity and mortality, it is vital that testing be performed in areas of greatest need, where more severe cases are expected. The goal of this analysis is to evaluate COVID-19 testing data in NYC relative to risk factors for COVID-19 disease severity and demographic characteristics of NYC neighborhoods.

**Methods:**

COVID-19 testing and the racial/ethnic composition of NYC Zip Code Tabulation Areas (ZCTA) were obtained from the NYC Coronavirus data repository and the American Community Survey, respectively. The prevalence of neighborhood-level risk factors for COVID-19 severity according to the Centers for Disease Control and Prevention criteria for risk of severe illness and complications from COVID-19 were used to create a ZCTA-level risk index. Poisson regressions were performed to study the ratio of total tests relative to the total ZCTA population and the proportion of positive tests relative to the total tests performed over time.

**Results:**

From March 2nd-April 6th, the total tests/population (%) was positively associated with the proportion of white residents (IRR_adj_: 1.0003, 95% CI: 1.0003–1.0004) and the COVID risk index (IRR_adj_: 1.038, 95% CI: 1.029–1.046). The risk index (IRR_adj_: 1.017, 95% CI: 0.939–1.101) was not associated with total tests performed from April 6th-May 12th, and inversely associated from May 12th-July 6th (IRR_adj_: 0.862, 95% CI: 0.814–0.913). From March 2nd-April 6th the COVID risk index was not statistically associated (IRR_adj_: 1.010, 95% CI: 0.987–1.034) with positive tests/total tests. From April 6th-May 12th, the COVID risk index was positively associated (IRR_adj_: 1.031, 95% CI: 1.002–1.060), while from May 12th-July 6th, the risk index was inversely associated (IRR_adj_: 1.135, 95% CI: 1.042–1.237) with positivity.

**Conclusions:**

Testing in NYC has suffered from the lack of availability in high-risk populations, and was initially limited as a diagnostic tool for those with severe symptoms, which were mostly concentrated in areas where vulnerable residents live. Subsequent time periods of testing were not targeted in areas according to COVID-19 disease risk, as these areas still experience more positive tests.

## Background

The clinical syndrome caused by Coronavirus (COVID-19) continues to be a global problem that requires a comprehensive and evolving public health strategy. There are currently 33 million confirmed cases and roughly 591,000 confirmed deaths in the US from COVID-19, as of June 2021 [1]. Since the first documented case in New York City (NYC) on March 1st, NYC became the epicenter of the infection and of mortality in the US [2]. Only recently have public health efforts of curbing transmission obtained some measurable success. However, the rampant number of cases and deaths in March through May was partly a consequence of lack of widespread COVID-19 testing, which was implemented relatively late and at a very slow pace. In March through early May, the New York City Department of Health (NYCDOH) discouraged those with mild and moderate symptoms from being tested [3]. In February, some initial test kits from the CDC were reported to have inadequate negative controls, and these test kits were not reliable [4]. Within NYC, all tests reported by the NYCDOH rely on the polymerase chain reaction (PCR) test to confirm COVID-19 infection [4]. Additionally, there was a slow adoption of non-pharmaceutical interventions and lockdowns throughout the US and NYC, including increased protections for vulnerable populations, inadequate or delayed implementation of masks, and social distancing guidelines [5, 6]. While testing should be performed to inform contact tracing in order to prevent COVID-19 spread, it was initially utilized as a diagnostic tool due to lack of personnel and testing infrastructure. Therefore, testing was initially not utilized for contact tracing, and a lack of tests and unclear guidelines led to testing being performed only in cases presenting with likely symptoms.

Throughout the United States, it has been noted that racial health disparities exist with respect to COVID-19 infection, morbidity, and mortality rates [7–12]. In general, people of color and ethnic minorities are disproportionately more likely to belong to lower socioeconomic sections of the population, face economic inequality, have underlying health conditions that influence COVID-19 outcomes, have diminished access to healthcare, potentially live in more crowded areas, and represent a larger proportion of essential workers [7]. Current data in New York State (NYS) suggests that Hispanic and black populations have higher morality [13], and in NYC specifically, have higher age-adjusted rates of non-hospitalized, hospitalized-non fatal, and hospitalized fatal COVID-19 cases than white residents [13].

The role of comorbidities in the clinical course and outcomes of COVID-19 infections have been discussed and documented globally [14], in the US as a whole [15], and in New York [16]. One study encompassing all COVID-19 patients in NYS commented that asthma, hypertension and diabetes were the most commonly presented comorbidities [16]. A retrospective analysis of patients from NYC and the greater NYC area indicated that 88% of hospitalized COVID-19 patients had ≥1 comorbidity, and that obesity, diabetes, and hypertension were common in this sample, although race was not taken into account in the analysis [17]. Given the negative effect of comorbidities on COVID-19 morbidity and mortality, it is vital that COVID-19 testing and early detection be performed in areas of greatest need where the prevalence of comorbidities is high.

In order to study if testing was administered effectively and in areas of need in NYC, such as those areas where the most vulnerable subjects live, we analyzed prevalence testing data provided by the NYCDOH at three-time intervals: March 2nd to April 6th, April 6th to May 12th, and May 12th to July 6th, and examined this in conjunction with data on the geographic distribution of risk factors for COVID-19 severity in NYC. The objectives of this analysis are: 1) to determine changes in the distribution of COVID-19 tests, COVID-19 positive tests, the proportion of the cumulative COVID-19 tests performed relative to the total Zip Code Tabulation Area (ZCTA) population, as well as the proportion of positive / total tests according to ZCTA over time, 2) determine if testing and positive testing was associated with the presence of COVID-19 risk factors according to ZCTA, and if this association changed over time. We hypothesized that testing originally was not performed in areas of greatest need, but has shifted to accommodate this need.

## Methods

This was an ecological study of COVID-19 prevalence testing and neighborhood characteristics with a cross-sectional analysis at three time points: March 2nd to April 6th, April 6th to May 12th, and May 12th to July 6th. The first window represents the time when the first COVID-19 case was recorded in New York and includes the following month encompassing the first fatalities, closures of schools, bars, restaurants, and the beginning of non-essential workers staying home [6]. The next period represents the extension of widespread closures and home-orders by the Governor and extended responses to COVID-19. The last period ending on July 6th represents when Phase 3 of reopening started in NYC. The main outcomes of this study were the ratio of the cumulative COVID-19 tests performed relative to the total ZCTA population and the proportion of positive tests per number of tests, measured during these time windows. The main predictor of interest was the COVID severity risk at the ZCTA level. There were 177 ZCTAs in NYC reporting COVID-19 testing data.

### COVID severity risk index

Risk factors in this analysis were selected according to the CDC criteria for risk of severe illness and complications from COVID-19 [18], as described and implemented previously [19–21]. NYC census-tract level estimates of various comorbidities were downloaded from the 500 Cities Project [22] and the 2017 Behavioral Risk Factor Surveillance System (BRFSS) [23]. Liver disease was approximated through hepatitis B and hepatitis C prevalence (2017 Communicable Disease Surveillance Data [24]), and alcohol related hospitalizations (New York State’s hospital discharge database [25]). Birth rates were downloaded from the New York City Department of Mental Health and Hygiene, while the New York City Environment and Health Data Portal [26] was queried for rates of heart attacks. Information on age, race, and ethnicity were downloaded from 2018 American Community Survey 5-year estimates [27]. All data were converted to ZIP code, using census [28] and NYC DOH [29] crosswalks. Each risk factor had full coverage across NYC, with the exception of the birth rate, Hepatitis B, and Hepatitis C variables, which were missing for 5 ZCTAs that were excluded from this analysis. Across all ZCTAs, each risk factor was scored from 1 to 4 based on quartiles, where higher scores represent higher prevalence. Individual scores were summed across risk factors to create an overall risk score for each ZCTA, with higher values corresponding to areas with greater risk. The overall risk score was analyzed in quartiles for all NYC ZCTAs.

### COVID-19 tests by Zip Code Tabulation Area

The number of COVID-19 tests performed and the number of positive COVID-19 tests were downloaded from the NYC Coronavirus (COVID-19) data repository hosted by the NYCDOH over three time periods: March 2nd to April 6th, April 6th to May 12th, and May 12th to July 6th. The data repository began on March 2nd. The COVID-19 positive test prevalence, COVID-19 test prevalence, the risk index, and the racial and ethnic composition by ZCTA were geographically mapped.

### Statistical analysis

The geographic unit of analysis was the ZCTA and testing data refers to person’s ZCTA of residence. Wilcoxon rank-sum tests were performed to assess differences in risk index components, racial composition, Hispanic composition, positive tests, and total tests according to risk index quartiles across NYC ZCTAs. Univariate and multivariable Poisson regressions were performed to predict the ratio of the cumulative tests performed relative to the total ZCTA population utilizing the risk index, the median age, and the racial and ethnic composition at the ZCTA level as predictors. This was performed for time windows from March 2nd to April 6th, April 6th to May 12th, and from May 12th to July 6th. In these models a Pearson scaling factor was used to correct for overdispersion, and a log (population) term was used as an offset. Poisson regression was also performed to predict the proportion of positive tests per total number of tests performed according to the risk index, with the median age, and the racial and ethnic composition of the ZCTA as predictors for the same time windows. Predictors were rescaled to units of 10 for these models. All tests of statistical significance were two sided, at α = 0.05. All analyses were performed in SAS v9.4 and RStudio v1.4.1106.

## Results

### COVID severity risk index

There were statistically significant increases (*p* <  0.05) in the prevalence of many risk factors (asthma, kidney disease, hypertension, heart disease, obesity, COPD, diabetes, Hepatitis C, Hepatitis B, residents aged ≥65 years, birth rate, alcohol hospitalizations, Hispanic residents) across risk index quartiles (Table 1). However, the proportion white residents (*p* <  0.0001) decreased, and the prevalence of cancer (excluding skin cancer) (*p* = 0.1333) and the median age (*p* = 0.1326) were similar across risk quartiles.

**Table 1:** COVID risk index components, median age, and the racial and Hispanic composition of NYC ZCTAs according to quartiles of the risk index

Variable	Quartile 1 (lowest risk score; 21.46–40.26) mean (SD)	Quartile 2 (40.27–48.15) mean (SD)	Quartile 3 (48.16–55.01) mean (SD)	Quartile 4 (highest risk score; 55.02–73.84) mean (SD)	*p*-value
Median age (years)	35.4 (3.31)	37.64 (3.81)	36.71 (4.26)	36.73 (6.23)	0.1326
Proportion white residents (%)	66.68 (15.40)	45.89 (21.26)	36.78 (23.53)	32.21 (26.81)	< 0.0001
Proportion Hispanic residents (%)	17.12 (12.40)	29.13 (18.53)	25.67 (18.82)	34.52 (22.98)	0.0004
Obesity prevalence (%)	17.87 (5.01)	22.31 (4.24)	26.53 (4.69)	29.72 (5.98)	< 0.0001
Kidney disease prevalence (%)	2.17 (0.49)	2.84 (0.27)	3.2 (0.26)	3.67 (0.48)	< 0.0001
Hypertension prevalence (%)	20.36 (4.00)	26.43 (2.15)	30.37 (3.13)	33.60 (3.41)	< 0.0001
Heart Disease prevalence (%)	3.75 (1.01)	5.25 (0.42)	5.76 (0.59)	6.49 (0.81)	< 0.0001
Diabetes prevalence (%)	6.87 (2.27)	10.48 (1.58)	11.78 (1.40)	13.13 (2.17)	< 0.0001
COPD prevalence (%)	3.78 (1.11)	5.17 (0.62)	6.04 (0.72)	6.75 (0.90)	< 0.0001
Cancer (except skin) prevalence (%)	5.02 (1.14)	5.49 (1.03)	5.55 (0.94	5.79 (1.53)	0.1333
Asthma prevalence (%)	8.46 (0.74)	8.92 (0.95)	10.11 (1.34)	10.80 (1.26)	< 0.0001
Alcohol Hospitalizations Prevalence (%)	1.71 (1.02)	1.36 (0.56)	1.51 (0.77)	2.26 (1.20)	0.0005
Birth rate (%)	1.25 (0.37)	1.16 (0.32)	1.26 (0.32)	1.30 (0.23)	0.0325
Hepatitis C prevalence (%)	0.05 (0.02)	0.05 (0.01)	0.05 (0.01)	0.08 (0.03)	< 0.0001
Hepatitis B prevalence (%)	0.06 (0.07)	0.09 (0.09)	0.08 (0.07)	0.07 (0.04)	< 0.0001
Proportion ≥ 65 years old (%)	12.3 (4.7)	14.88 (4.09)	14.51 (3.72)	15.73 (5.95)	0.0378

The racial and Hispanic composition of each ZCTA were obtained from the 2018 American Community Survey 5-year estimates. Unless otherwise noted, all variables are reported per hundreds of residents. Each variable was measured at the ZCTA level

### Geography of COVID-19 testing

**Fig. 1:**
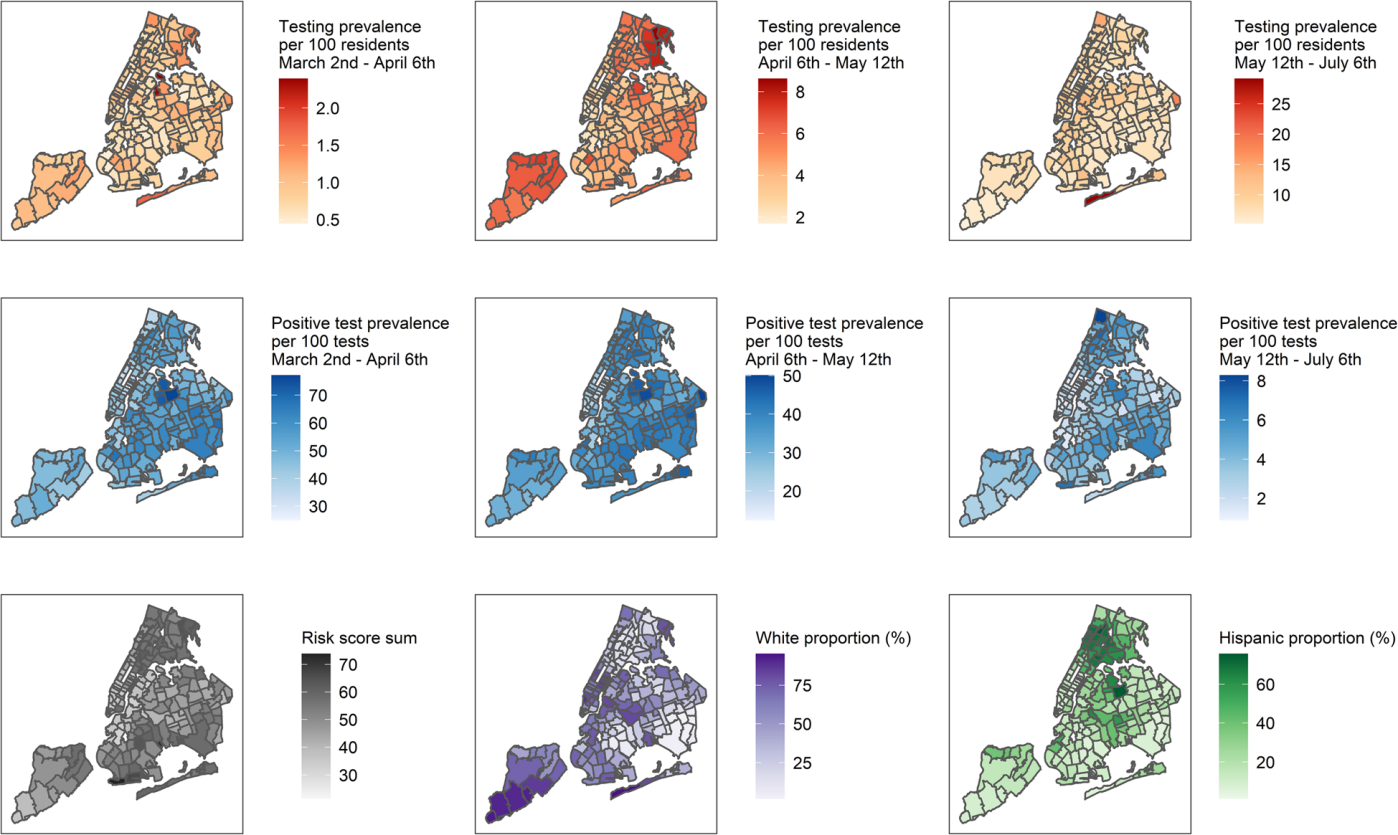
Distribution of the testing prevalence per 100 residents from March 2nd to April 6th (top-left), April 6th to May 12th (top-center), and from May 12th to July 6th (top-right), positive test prevalence per 100 tests from March 2nd to April 6th (middle-left), April 6th to May 12th (middle-center), and from May 12th to July 6th (middle-right), quartiles of the COVID severity risk index (bottom-left), the proportion white residents (bottom-center), and the Hispanic composition (%) (bottom-right) across New York City Zip Code Tabulation Areas. Proportion total tests / population (%) displayed in hundreds of residents

**Fig. 2:**
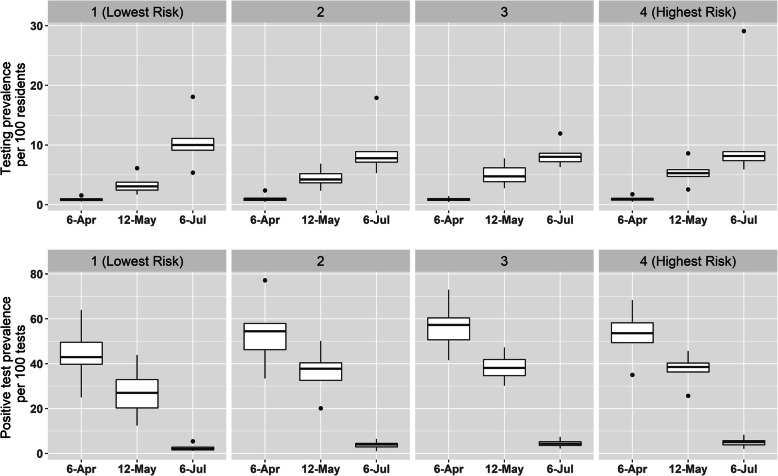
Distribution of the testing prevalence per 100 residents (top) and the positive test prevalence per 100 tests (bottom) from March 2nd to April 6th, April 6th to May 12th, and from May 12th to July 6th, across quartiles of the COVID severity risk index

The distribution of total tests/population and positive tests/total tests in each time window were mapped according to ZCTA, as well as the risk index and the racial and Hispanic proportion (Fig. 1). From March 2nd to April 6th, the ratio of total tests / population was similar (*p* = 0.5947) and the proportion of positive tests / total tests (*p* < .0001) increased across increasing quartiles of the risk index (Fig. 2). From April 6th to May 12th, the ratio of total tests / population (*p* <  0.0001) and the proportion of positive tests / total tests (*p* <  0.0001) increased across increasing quartiles of the risk index. From May 12th to July 6th, the ratio of total tests / population (p <  0.0001) slightly decreased and the proportion of positive tests / total tests (p <  0.0001) increased across increasing quartiles of the risk index.

### Analyzing COVID-19 testing

In the time frame from March 2nd to April 6th, the ratio of total tests/population significantly increased with the increasing proportion of white residents in the ZCTA (IRR_adj_: 1.0003, 95% CI: 1.0003–1.0004), Hispanic composition (IRR_adj_: 1.0001, 95% CI: 1.0001–1.0002), median age of the ZCTA (IRR_adj_: 1.0007, 95% CI: 1.0005–1.0009) and the risk index (IRR_adj_: 1.038, 95% CI: 1.029–1.046) (Table 2). From April 6th to May 12th and May 12th to July 6th, there were no significant association between the proportion of white residents or the Hispanic composition with testing, and a positive and statistically significant association with the median age of the ZCTA From April–May (IRR_adj_: 1.005, 95% CI: 1.003–1.007) and May–July (IRR_adj_: 1.004, 95% CI: 1.002–1.005). Increasing risk index quartiles was positively associated with testing from March–April (IRR_adj_: 1.038, 95% CI: 1.029–1.046), not significant from April–May (IRR_adj_: 1.017, 95% CI: 0.939–1.101), and inversely associated with testing from May–July (IRR_adj_: 0.862, 95% CI: 0.814–0.913). In the time frame from March 2nd to April 6th, the white proportion, Hispanic proportion, and risk index (IRR_adj_: 1.010, 95% CI: 0.987–1.034) were not significantly associated with the ratio of positive tests to total tests in the ZCTA. From April 6th to May 12th, an increasing median age (IRR_adj_: 1.001, 95% CI: 1.001–1.002) and the risk index (IRR_adj_: 1.031, 95% CI: 1.002–1.060) were statistically significantly associated with increasing positive testing. From May 12th to July 6th, the risk index remained significantly associated (IRR_adj_: 1.135, 95% CI: 1.042–1.237) with positive testing.

**Table 2:** Predictors of testing prevalence and positive test prevalence over time

	Total Tests / Population^a^					
Timeframe	March 2nd to April 6th		April 6th to May 12th		May 12th to July 6th	
	IRR_unadj_ (95% Confidence Limits)	IRR_adj_ (95% Confidence Limits)	IRR_unadj_ (95% Confidence Limits)	IRR_adj_ (95% Confidence Limits)	IRR_unadj_ (95% Confidence Limits)	IRR_adj_ (95% Confidence Limits)
White residents (%)	1.0003 (1.0003–1.0003)	1.0003 (1.0003–1.0004)	1.000 (0.999–1.000)	0.999 (0.999–1.000)	1.0003 (1.0003–1.0003)	1.000 (0.999–1.000)
Hispanic composition (%)	0.999 (0.999–1.000)	1.0001 (1.0001–1.0002)	0.999 (0.999–1.000)	1.000 (0.999–1.000)	0.999 (0.998–0.999)	1.000 (0.999–1.000)
Median age (years)	1.001 (1.000–1.001)	1.0007 (1.0005–1.0009)	1.004 (1.002–1.006)	1.005 (1.003–1.007)	1.004 (1.003–1.005)	1.004 (1.002–1.005)
COVID risk index quartiles	1.002 (0.996–1.009)	1.038 (1.029–1.046)	1.038 (0.971–1.109)	1.017 (0.939-1.101)	0.834 (0.797–0.875)	0.862 (0.814–0.913)
	Positive Tests / Total Tests^b^					
Timeframe	March 2nd to April 6th		April 6th to May 12th		May 12th to July 6th	
	IRR_unadj_ (95% Confidence Limits)	IRR_adj_ (95% Confidence Limits)	IRR_unadj_ (95% Confidence Limits)	IRR_adj_ (95% Confidence Limits)	IRR_unadj_ (95% Confidence Limits)	IRR_adj_ (95% Confidence Limits)
White residents (%)	1.000 (0.999–1.000)	0.999 (0.999–0.999)	0.999 (0.999–1.000)	0.999 (0.999–0.999)	0.999 (0.999–0.999)	0.999 (0.998–0.999)
Hispanic composition (%)	1.000 (1.000–1.000)	1.000 (0.999–1.000)	1.001 (1.000–1.001)	1.000 (1.000–1.000)	1.001 (1.001–1.002)	1.000 (1.000–1.000)
Median age (years)	0.999 (0.999–1.000)	0.999 (0.999–1.000)	1.000 (0.999–1.000)	1.001 (1.001–1.002)	0.998 (0.997 – 0.999)	1.001 (0.998–1.003)
COVID risk index quartiles	1.056 (1.037–1.077)	1.010 (0.987–1.034)	1.108 (1.083–1.133)	1.031 (1.002–1.060)	1.248 (1.164–1.338)	1.135 (1.042–1.237)

^a^Poisson regression performed, adjusted with a Pearson scaling factor to correct for overdispersion, log (population) used as an offset

^b^Poisson regression performed

Results shown for unadjusted and adjusted models. Models were adjusted for all variables shown. The total tests / population was calculated per hundred residents. A larger risk index quartile represents higher risk. Results for white residents (%), Hispanic composition (%) and Median age are reported in units of 10. IRR: incidence rate ratio

## Discussion

This analysis comments on spatial and temporal variation in NYC COVID-19 testing across multiple timeframes. Here we report that roughly 10 days after the complete shutdown of NYC and NYS, COVID-19 testing was performed in areas with increased risk of COVID severity and in areas with a greater white racial composition and Hispanic composition. This reflects how initial NYCDOH testing was recommended in cases presenting with severe symptoms of COVID-19, due to testing shortages. Therefore, this result is an indirect measure of the prevalence of serious symptoms and disease in the population, as it is used as a diagnostic test rather than a preventative tool [3]. Certain barriers to testing likely existed, such as access to testing, financial means to pay for testing, and insurance type, so disparities in testing existed [30, 31]. From April 6th to May 12th, testing was not focused in these at-risk areas, where a large number of residents report numerous comorbidities and pre-existing conditions, and instead became more widespread rather than targeted to those who presented with severe symptoms. The proportion of positive tests was also higher from April 6th to May 12th in NYC ZCTAs with the greatest risk, supporting the notion of further performing widespread testing in these geographic areas of need. The continuation of this trend is represented in the May 12th to July 6th period, where testing became even more available and widespread.

Previous literature has emphasized geographic variation in COVID-19 tests, hospitalizations and deaths across NYC, although this information was analyzed by borough [8]. While this existing analysis is important, a finer geographic unit of analysis is needed as NYC boroughs are highly heterogeneous, and COVID-19 testing vulnerability is not uniformly distributed. The same can be also said about the population characteristics, as socioeconomic status, income, and education are notably heterogeneous at the borough-level and vary greatly by ZCTA.

Despite the observation that more COVID-19 tests overall were recently performed and extended to residents of vulnerable areas, there remain pockets of vulnerability in communities where testing has not sufficiently increased, such as the Bronx, Queens, and Brooklyn (Fig. 1). Another aspect to note is that racial disparities in access to testing remain as of today, despite the need for testing in communities that experience a large number of essential workers living in crowded realities [32].

### Limitations

One limitation of this analysis is that comorbidity information was collected in 2017 and 2016, although this was the most recent public information available. Five ZCTAs did not contain the full risk index information and were excluded from this analysis. We made the assumption that the prevalence of comorbidities has not changed significantly over a 3–4 year period. If individual level data on comorbidities were available, the most appropriate comorbidity index could be employed [33]. This would avoid an ecological fallacy, and future studies could provide individual-level conclusions about comorbidies and risk. The variables included in the risk index are self-reported, and thus could represent an imprecise estimate. Likewise, COVID testing data are also likely to be affected by underreporting [34]. These were contributing reasons why windows were chosen to present prevalence of testing and positivity rather than a single point in time.

This was an ecological study that utilized aggregate ZCTA-level data, which limits the ability to draw individual-level conclusions. For instance, we cannot comment on the interaction between being non-white and having a high risk index score on the likelihood of testing or of a positive test, knowledge that would help to identify most vulnerable populations. There are likely more individual level factors that may influence COVID-19 testing that could not be taken into account here. However, this analysis attempts to monitor how NYC has addressed the well-known disparities in COVID-19 testing, and can inform the reasons why the decline in new cases and hospitalization rates has been slower than expected.

## Conclusions

The results suggest that from April 6th to May 12th, testing distribution in NYC has suffered from the lack of availability of sufficient testing, and was limited as a diagnostic tool in those with severe symptoms, which were mostly concentrated in areas where vulnerable residents live, as these areas have higher proportions of positive tests, comorbidities and pre-existing conditions. Further periods of widespread testing were still not targeted in areas according to COVID risk, as areas at greater risk of COVID severity still experience more positive tests. To prepare for continued waves of COVID-19 in NYC and mitigate the transmission of COVID-19, continued widespread diagnostic testing is needed, especially in vulnerable and minority communities.

## Data Availability

Data used in this analysis is available for open public access at https://github.com/nychealth/coronavirus-data. Individual data was not used.

## Abbreviations

ACS: American Community Survey
NYC: New York City
NYS: New York State
NYCDOH: New York City Department of Health
ZCTA: Zip Code Tabulation Area

## References

[1] World Health Organization. (2021). Coronavirus disease 2019 (COVID-19) 8 June 2021 Weekly Epidemiological Update. Retrieved from https://www.who.int/publications/m/item/weekly-epidemiological-update-on-covid-19%2D%2D-8-june-2021

[2] Dong E, Du H, Gardner L (2020). An interactive web-based dashboard to track COVID-19 in real time. Lancet Infect Dis.

[3] NYC coronavirus disease 2019 (COVID-19) data. New York City Department of Health. https://github.com/nychealth/coronavirus-data. Updated daily.

[4] Patel, Neel (2020). Why the CDC botched its coronavirus testing. MIT technology review. Retrieved from https://www.technologyreview.com/2020/03/05/905484/why-the-cdc-botched-its-coronavirus-testing/

[5] Shear MD, Goodnough a, Kaplan S, fink S, Thomas K, and Weiland N. the lost month: how a failure to test blinded the US to COVID-19. The New York times. https://www.nytimes.com/2020/03/28/us/testing-coronavirus-pandemic.html. Published March 28, 2020. Updated April 1, 2020. Accessed 13 April 2020.

[6] Press Office of Governor Andrew W Cuomo. Governor Cuomo Sings the ‘New York State on PAUSE’ Executive Order. New York State. https://www.governor.ny.gov/news/governor-cuomo-signs-new-york-state-pause-executive-order. Published March 20, 2020. Accessed 3 Sept 2020.

[7] Chowkwanyun M, Reed AL (2020). Racial health disparities and Covid-19—caution and context. N Engl J Med.

[8] Wadhera RK, Wadhera P, Gaba P, Figueroa JF, Maddox KEJ, Yeh RW, Shen C (2020). Variation in COVID-19 hospitalizations and deaths across New York City boroughs. JAMA..

[9] Lieberman-Cribbin W, Tuminello S, Flores RM, Taioli E (2020). Disparities in COVID-19 testing and positivity in New York City. Am J Prev Med.

[10] Karaye IM, Horney JA (2020). The impact of social vulnerability on COVID-19 in the US: an analysis of spatially varying relationships. Am J Prev Med.

[11] Nelson A (2002). Unequal treatment: confronting racial and ethnic disparities in health care. J Natl Med Assoc.

[12] Yancy CW. COVID-19 and African Americans. JAMA. 2020. 10.1001/jama.2020.6548 32293639

[13] New York State Department of Health (2020). NYS Fatalities by County. Retrieved from https://covid19tracker.health.ny.gov/views/NYS-COVID19-Tracker/NYSDOHCOVID-19Tracker-Fatalities?%3Aembed=yes&%3Atoolbar=no&%3Atabs=n#/views/NYS%2dCOVID19%2dTracker/NYSDOHCOVID%2d19Tracker%2dMap?%253Aembed=yes&%253Atoolbar=no). Accessed 04/28/2020.

[14] Jordan R E, Adab P, Cheng KK. Covid-19: risk factors for severe disease and death. BMJ. 2020;368:m1198. 10.1136/bmj.m1198. 10.1136/bmj.m1198 32217618

[15] COVID C, Team R (2020). Severe outcomes among patients with coronavirus disease 2019 (COVID-19)—United States, February 12–March 16, 2020. MMWR Morb Mortal Wkly Rep.

[16] Rosenberg ES, Dufort EM, Blog DS, Hall EW, Hoefer D, Backenson BP (2020). COVID-19 testing, epidemic features, hospital outcomes, and household prevalence, New York state—March 2020. Clin Infect Dis.

[17] Richardson S, Hirsch JS, Narasimhan M, Crawford JM, McGinn T, Davidson KW, et al. Presenting characteristics, comorbidities, and outcomes among 5700 patients hospitalized with COVID-19 in the New York City area. JAMA. 2020. 10.1001/jama.2020.6775 PMC7177629 32320003

[18] Centers for Disease Control and Prevention. Coronavrius Disease 2019 (COVID-19) People Who Are at Increased Risk for Severe Illness. Updated June 25, 2020. https://www.cdc.gov/coronavirus/2019-ncov/need-extra-precautions/people-at-increased-risk.html?CDC_AA_refVal=https%3A%2F%2Fwww.cdc.gov%2Fcoronavirus%2F2019-ncov%2Fneed-extra-precautions%2Fpeople-at-higher-risk.html

[19] Raifman MA, Raifman JR (2020). Disparities in the population at risk of severe illness from COVID-19 by race/ethnicity and income. Am J Prev Med.

[20] Razzaghi H, Wang Y, Lu H, Marshall KE, Dowling NF, Paz-Bailey G, Twentyman ER, Peacock G, Greenlund KJ (2020). Estimated county-level prevalence of selected underlying medical conditions associated with increased risk for severe COVID-19 illness—United States, 2018. Morb Mortal Wkly Rep.

[21] Lieberman-Cribbin W, Alpert N, Flores R, Taioli E (2021). A risk index for COVID-19 severity is associated with COVID-19 mortality in nEw York City. BMC Public Health.

[22] Centers for Disease Control and Prevention, National Center for Chronic Disease Prevention and Health Promotion, Division of Population Health. 500 Cities Project Data [online]. 2018 [Accessed Oct 23, 2018]. URL: https://www.cdc.gov/500cities.

[23] Centers for Disease Control and Prevention (CDC). Behavioral Risk Factor Surveillance System Survey Data. Atlanta, Georgia: U.S. Department of Health and Human Services, Centers for Disease Control and Prevention, [2017].

[24] New York City Department of Health and Mental Hygiene. EpiQuery. Accessed July 29, 2020. Available at https://nyc.gov/health/epiquery

[25] New York State Department of Health. Statewide Planning and Research Cooperative System (SPARCS). https://www.health.ny.gov/statistics/sparcs/

[26] New York City Department of Health. Environment & Health Data Portal. Heart Attack Hospitalizations. http://a816-dohbesp.nyc.gov/IndicatorPublic/VisualizationData.aspx?id=90,4466a0,13,Summarize.

[27] U.S. Census Bureau; American Community Survey, 2018 American Community Survey 5-Year Estimates, Tables B02001 and B03003. [Accessed 04/28/2020].

[28] United States Census Bureau. Relationship Files. Available at: https://www2.census.gov/geo/docs/maps-data/data/rel/zcta_tract_rel_10.txt

[29] New York City United Hospital Fund Codes. Available at https://www1.nyc.gov/assets/doh/downloads/pdf/ah/zipcodetable.pdf

[30] McElfish PA, Purvis R, James LP, Willis DE, Andersen JA (2021). Perceived barriers to COVID-19 testing. Int J Environ Res Public Health.

[31] Kim EJ, Marrast L, Conigliaro J (2020). COVID-19: magnifying the effect of health disparities. J Gen Intern Med.

[32] Centers for Disease Control and Prevention (2020). COVID-19 in Racial and Ethnic Minority Groups. Retrieved from https://www.cdc.gov/coronavirus/2019-ncov/need-extra-precautions/racial-ethnic-minorities.html

[33] Ou HT, Mukherjee B, Erickson SR, Piette JD, Bagozzi RP, Balkrishnan R (2012). Comparative performance of comorbidity indices in predicting health care-related behaviors and outcomes among Medicaid enrollees with type 2 diabetes. Population health management.

[34] Wu SL, Mertens AN, Crider YS, Nguyen A, Pokpongkiat NN, Djajadi S, Seth A, Hsiang MS, Colford JM, Reingold A, Arnold BF. Substantial underestimation of SARS-CoV-2 infection in the United States. Nat Commun 2020;11(1):1–0, 4507, DOI: 10.1038/s41467-020-18272-4. 10.1038/s41467-020-18272-4 PMC7481226 32908126

